# Royal London Ophthalmic Hospital

**Published:** 1888-01-21

**Authors:** Robert Newstead

**Affiliations:** Secretary


### ROYAL LONDON OPHTHALMIC HOSPITAL.

I wish to correct in one respect your excellent report of the Christmas entertainment given at this hospital on Wednesday evening, January 4th. The arrangements were especially made by Mr. E. Treacher Collins, the senior house surgeon, to whom the credit is due for their excellence; and, in fact, the concert was given by the *resident* staff, which does not include yours obediently,

January 16th, 1888. Robert Newstead, Secretary.

